# Reference of serum insulin level and prevalence of insulin resistance of Korean children and adolescents

**DOI:** 10.1186/1687-9856-2013-S1-P99

**Published:** 2013-10-03

**Authors:** Jung Sub Lim, Ju Hee Seo, Jun Ah Lee, Dong Ho Kim

**Affiliations:** 1 Department of Pediatrics, Korea Cancer Center Hospital, Seoul,139–706, Korea

**Keywords:** Insulin resistance, Homeostasis model assessment, Metabolic syndrome, Korean children adolescent, National Health, Nutrition Examination Survey

## Objective

This study was to examine the distribution of insulin and homeostasis model assessment of insulin resistance (HOMA-IR) and decide insulin resistance cut off of Korean children and adolescents.

## Research Design and Methods

Data from 2,716 subjects (1,421 male, 1,295 female; age range, 10-20 years) in Korea National Health and Nutrition Examination Survey IV (2007–2009) were evaluated. The reference values of insulin and HOMA-IR of normal weight subjects were made according to sex, age and weight status. Insulin resistance was defined as HOMA-IR > 95 percentile. The odds ratios of metabolic syndrome and its components were assessed based on insulin resistance state.

## Results

Insulin and HOMA-IR values appear to peak at age 13-14 years in male and age 12-13 years in female subjects. Female had lower fasting glucose and higher insulin (P = 0.049) than male. Thus, HOMA-IR between sex was not different (P = 0.257). Overweight and obese subjects had higher HOMA-IR compared with subjects of normal weight (3.83 [95% CI 3.64–4.02], 5.16 [95% CI 4.70–5.62], and 2.66 [2.62–2.71], respectively). The prevalence of insulin resistance in total subjects was 9.7% (male; 10.9 %, vs. female; 8.6 %). The prevalence of insulin resistance in normal-weight, overweight and obese subjects were 4.7%, 25.6%, and 47.1% respectively. Subject with insulin resistance had more metabolic syndrome (odds ratios, 18.33 [95% CI 9.62–34.94]) and its components.

## Conclusions

Insulin and HOMA-IR values vary depending on sex, age, and weight status. Obesity is the most important risk factor for insulin resistance, but number of insulin resistance subject in normal-weight subject were compatible to those. This information may be useful in not only Korean but also Asian planning programs for the prevention of type 2 diabetes from childhood.

